# Assessment of stress and anxiety in mice with colorectal cancer submitted to physical exercise

**DOI:** 10.1590/acb370508

**Published:** 2022-08-12

**Authors:** Udenilson Nunes da Silva, Amanda Boutrik, Alessandra de Figueiredo Gonçalves, Marcelo Barbosa Neves, Gabriela Rodrigues Alves, Letícia Silva Fagundes, Antônio Carlos de Abreu, Ricardo Dutra Aydos, Rondon Tosta Ramalho

**Affiliations:** 1 Graduate Student. Universidade Federal do Mato Grosso do Sul – Faculty of Medicine – Campo Grande (MS), Brazil.; 2 Fellow master degree. Universidade Federal do Mato Grosso do Sul – Postgraduate Program in Health and Development in the Midwest Region – Campo Grande (MS), Brazil.; 3 Fellow PhD degree. Universidade Federal do Rio de Janeiro – Postgraduate Program in Biological Sciences (Physiology) – Rio de Janeiro (RJ), Brazil.; 4 Fellow PhD degree. Universidade Federal do Mato Grosso do Sul – Postgraduate Program in Health and Development in the Midwest Region – Campo Grande (MS), Brazil.; 5 Full professor. Universidade Federal do Mato Grosso do Sul – Postgraduate Program in Health and Development in the Midwest Region – Campo Grande (MS), Brazil.

**Keywords:** Models, Animal, Colitis-Associated Neoplasms, Elevated Plus Maze Test, Exercise

## Abstract

**Purpose:**

To evaluate the effect of physical exercise on the behavior of rodents with colorectal cancer induced through the use of elevated plus maze.

**Methods:**

We used 40 male hairless mice induced to colorectal cancer, divided into five groups: G1) submitted to pre- and post-induction swimming; G2) pre- and post-induction ladder; G3) post-induction swimming; G4) post-induction ladder; G5) sedentary. At the end of the 14th week, the animals were submitted to the plus maze test.

**Results:**

The mean length of stay in the open arm for G1 was 4.17 ± 6.50; G2 37.52 ± 40.7; G3 85.84 ± 42.5; G4 32.92 ± 23.17; and G5 4.09 ± 4.43. In the closed arm, it was 264 ± 23.43 in G1, 187.60 ± 47.73 in G2, 147.50 ± 40.03 in G3, 182.00 ± 40.40 in G4, and in G5 235.36 ± 14.28. In the center, G1 remained 31.86 ± 20.18, G2 74.85 ± 28.37, G3 66.69 ± 19.53, G4 60.55 ± 10.46, and G5 60.55 ± 23.65.

**Conclusions:**

Aerobic exercise for seven weeks after tumor induction showed less impact on the behavior of the animals. On the other hand, it significantly increased the animals’ stress level when applied for 14 weeks before and after tumor induction.

## Introduction

Colorectal cancer (CRC) is the third most common cancer in men and the second most common in women. In 2018, there were about 1.9 million new cases worldwide^1^.

The therapeutic management of CRC is generally invasive and aggressive, involving surgery, radio, and chemotherapy. Among the adverse effects of the treatment, there is a reduction in quality of life, associated with stress, anxiety, and depression, so the literature highlights a prevalence of depression ranging from 1.6 to 57% and a prevalence of anxiety ranging from 1 to 47.2% in patients with CRC^2^^,^ 3.

Evidence such as Benatti and Pedersen^4^ shows the effect of physical exercise as an anti-inflammatory in various tissues and organs. Chronic inflammation is considered one of the main mechanisms for promoting and accelerating the development of neurodegenerative diseases, as well as neoplasms. This process mainly involves the continuous activity of various cytokines. Skeletal muscle can function as an endocrine organ due to its production of growth hormones and cytokines known as myokines, which are induced by a stimulus to physical exercises, such as the protein brain-derived neurotrophic factor (BDNF) producing actions in the brain, such as in neuroplasticity function, as well as presenting an antidepressant effect, generating improvement in the cognitive system. According to a study by Sartori *et al*.^5^, voluntary physical activity increases the levels of mature BDNF in the hippocampus of mice, supporting the effect of exercise on antidepressant effects.

The literature points to the existence of a relationship between stress, progression, the emergence of tumors, and the emergence of metastases, as a result of the triggering of neuroendocrine processes and deregulation of the immune system^6^. In addition, the literature points to an inverse relationship between physical activity and cancer risk^7^. Physical exercise reduces the risk of mortality from CRC by 38%. The effects of physical exercise on improving mood, reducing anxiety, stress, and depression, as well as certain advantages over cognition, have also been described^6^^,^ 7.

Although the literature evidences the ability of physical exercise to improve well-being and to reduce the risk of CRC, there are still few studies on the relationship between physical exercise acting as a possible factor in improving the adverse effects derived from CRC such as anxiety, stress, and depression. This issue becomes relevant since such behavioral factors affect neuroendocrine processes and the immune system and may contribute to the development/worsening of the disease. Therefore, this study aimed to evaluate the effect of physical exercise on the behavior of rodents with CRC induced through the use of the elevated plus maze test, a validated method to investigate the behavioral aspects of anxiety-like in rodents^8^.

## Methods

The study was developed at the Laboratory of Experimental Carcinogenesis of the Postgraduate Program in Health and Development in the Midwest Region of the Universidade Federal do Mato Grosso do Sul (UFMS), in the municipality of Campo Grande (MS), Brazil. All steps and procedures were carried out in accordance with the ethical principles established by the National Council for the Control of Animal Experiments (CONCEA) and filed with the Ethics Committee on the Use of Animals of UFMS No. 1.091/2019.

Thirty-nine male inbred HRS/J mice, known as hairless, were used as experimental model of infection. The animals were obtained from the central animal facility of the Center for Biological and Health Sciences (CCBS) of the UFMS.

Mice were aged 4-5 weeks and had an initial weight of 25 g. The animals were kept in collective cages (dimension 40 × 35 × 17 cm), holding four animals/cage. Boxes were housed on the same shelf with a height of 1 meter from the floor and exposure to light in the same way as all the cages, at a temperature of approximately 25 °C, with a light/dark cycle of 12 hours, receiving standard chow (Nuvital® CR1) and water *ad libitum*. They were acclimated to laboratory conditions for 14 days before the experiment. The animals were randomly divided into five groups:

- G1: animals submitted to swimming (aerobic exercise) before and after tumor induction (n = 8);
- G2: animals submitted to ladder training (resistance exercise) before and after tumor induction (n = 8);
- G3: animals submitted to swimming (aerobic exercise) after tumor induction (n = 8);
- G4: animals submitted to ladder training (resistance exercise) after tumor induction (n = 8);
- G5: animals induced to cancer and kept sedentary (n = 7).

### Physical exercise protocols

Exercise protocols were performed on alternate days.

### Aerobic physical exercise

For this activity, the practice of swimming was chosen. The animals were submitted to adaptation six days before the beginning of the protocols, and they performed exercises gradually.

It was carried out in a 29-L tank of heated water (31 ± 2 °C) with height of 27.6 cm and width of 33 cm. The animals swam in groups of four mice. The duration of the swimming sessions was gradually increased, starting at 10 minutes and increasing by 5 minutes every three weeks^9^.

### Resistance exercises

The vertical ladder resistance training model was used, which consists of the animals climbing with weights tied to their tails until they reach a compartment at the top of the ladder with the total height of 110 cm, with steps made of stainless-steel bar, the distance between the steps: 1 cm, Tilt 80º. There were ten repetitions of ascent to the top of the stairs with a 2-min rest between ascents. They were starting with an overload of 10% of body weight, G2 ending with 50% of body weight, and G4 ending with 50% of body weight in the 14th week^10^.

### Colitis and colorectal cancer induction

**Figure 1:**
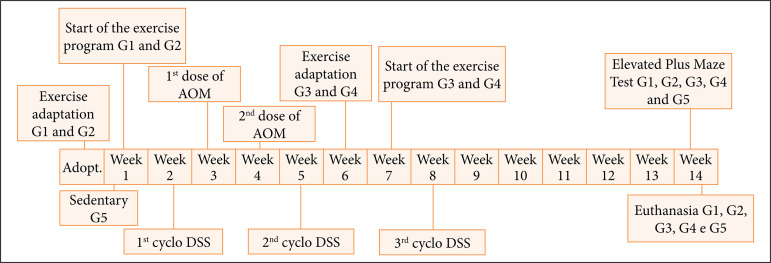
AOM: azoxymethane; DSS: dextran sulfate sodium. Experimental design: timeline.

For the induction of colitis, the mice were given, for three cycles of seven consecutive days, water containing dextran sulfate sodium (DSS) 2.5% (MP Biomedicals, Santa Ana, CA, United States of America), interspersed with two weeks of normal water, for the purpose of inducing intestinal inflammation (Fig. 1)^11^.

For cancer induction, all animals received two intraperitoneal injections (lower right quadrant of the abdomen) of azoxymethane (AOM – Sigma-Aldrich Laboratory), a total dose of 20 mg/kg, divided into two weeks, 10 mg/kg per week of AOM^11^.

### Cancer induction assessment

The presence of tumor in the colon was evaluated by obtaining fluorescent optical images within the near infrared (NIR) and X-ray images using the In-Vivo Xtreme/Bruker II system belonging to the Laboratory of Studies in Experimental Models of Diseases, after euthanasia through the presence of polyps and histological changes present in the distal colon.

To detect fluorescent images of the tumor, the animals received a dose of 0.334 mg/kg of the fluorescent biomarker IR-780 iodide Dye (Sigma Aldrich) intraperitoneally. After 12 hours, the animals were anesthetized in a 2%-isoflurane chamber and transferred to the In-Vivo Xtreme/Bruker II system, being kept anesthetized using isoflurane cones attached to the animal’s head. The fluorescent NIR images were captured using a 760 nm filter for excitation and 830 nm for emission. Fluorescent and X-ray images were captured simultaneously (and superimposed) for a perfect anatomical location.

### Elevated plus maze test: equipment setup

**Figure 2:**
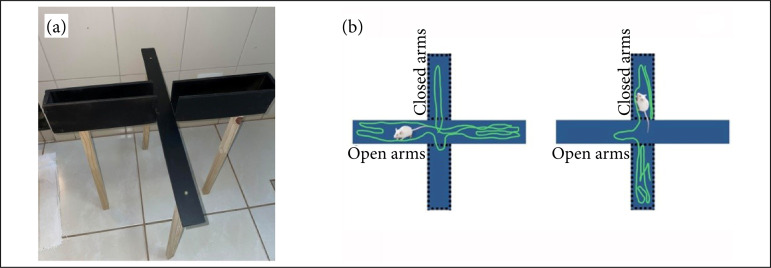
**(a)** Picture of the elevated plus maze used for testing mice; **(b)** typical mouse behavior withlow (*left*) and high (*right*) anxiety levels. The green trace demonstrates the animal’s movement. The leftrepresents more time in the open arms, while the right represents more time in the closed arms.

Our maze was made of wood with four arms, in the shape of a cross, two open arms without walls and two closed by 10 cm high walls; with 25 cm in length, 5 cm in width, and a central platform of 5 × 5 cm, raised 50 cm from the ground (Fig. 2a).

Each mouse was placed in the center of the platform, with the head directed towards one of the open arms; the time of permanence on the elevated plus maze was 5 min^12^. The sessions were recorded by a camcorder and analyzed by recording behaviors using the X-PloRat 3.0 program^13^.

Behavior records were performed regarding the length of stay and entry into the open and closed arms. The activity of the mice in the open arms reflects a conflict between the animal’s preference for the protected area (closed arms) and its innate motivation to explore a new environment. The increased length of stay and entries into the open arms signals are interpreted as decreased anxiety-like behavior (Fig. 2b)^8^.

### Statistical analysis

The elevated plus maze test results, expressed as a mean ± standard deviation, were submitted to the Kruskal-Wallis non-parametric test with Dunn’s post-test comparing the experimental groups’ physical exercise (G1, G2, G3, and G4) with the sedentary control group (G5). *P* values less than 5% (*p* < 0.05) were considered significant.

## Results

### Confirmation of tumor development

**Figure 3:**
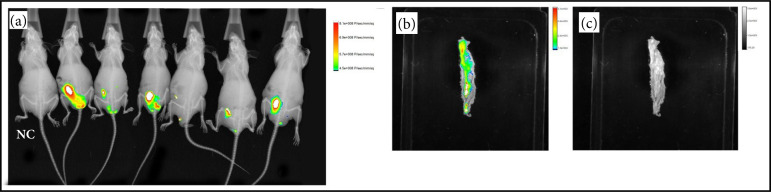
Images captured by the In-Vivo Xtreme/Bruker II system. **(a)** The animals superimposed by the fluorescence emitted by the colon (NC: negative control); **(b)** section photo of the colon superimposed by the fluorescence emitted by the polyps; **(c)** section photo of the distal colon.

**Figure 4:**
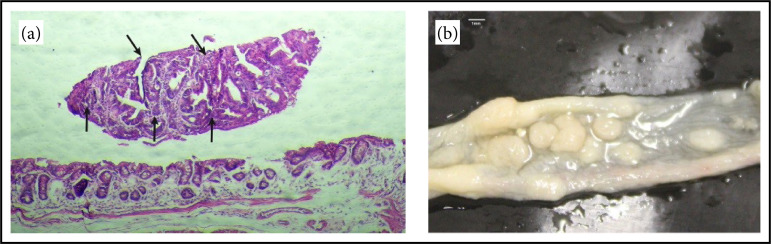
**(a)** Histological changes, arrows indicating colon adenoma;**(b)** polyps at the distal region of the colons.

Through the evaluation of fluorescence in the NIR, performed on the last day of the experiment, it was possible to observe the fluorescent labeling in all groups analyzed, indicating tumor development in the colons of the animals (Fig. 3). Those finds were confirmed after euthanasia by polyps and histological changes (such as aberrant crypts, adenomas, and low- and high-grade dysplasia) in hematoxylin and eosin at the distal region of the colons (Fig. 4).

### Elevated plus maze test

**Figure 5:**
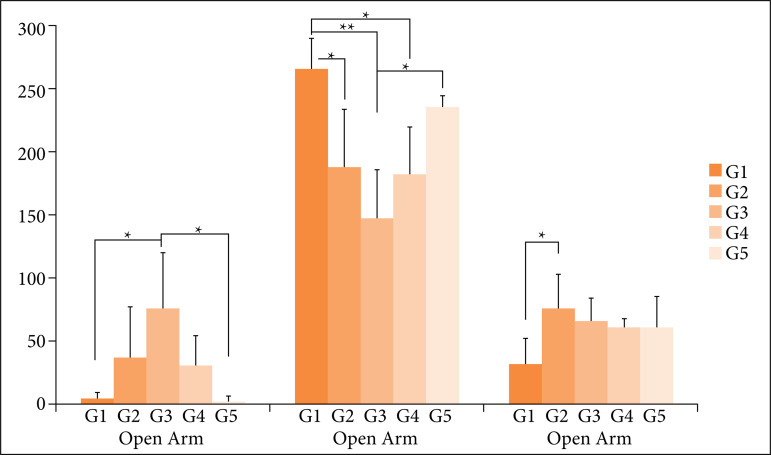
G1: aerobic group before and after tumor induction; G2: resistance exercise group before and after tumor induction; G3: aerobic exercise group after tumor induction; G4: resistance exercise group after tumor induction; G5: sedentary group; *significant difference (*p* < 0.05); **very significant difference (*p* < 0.0001). Time of permanence in the open, closed, and center arms during theelevated plus maze test of each group of animals submitted to the study.

The results of comparison between the exercise and sedentary experimental groups regarding the absolute time in seconds spent in open, closed, and center arms in the elevated plus maze test are described ahead and illustrated in Fig. 5.

The mean time (mean ± standard deviation) in the open arm for G1 was 4.171 ± 6.50; G2 37.52 ± 40.7; G3 85.84 ± 42.5; G4 32.92 ± 23.17; and G5 4.09 ± 4.43. There was a significant difference in the open arms between G1 and G3 (*p* < 0.05) and also between G3 and G5 (*p* < 0.05).

In the closed arm, the meantime in G1 was 264 ± 23.43; G2 187.60 ± 47.73; G3 147.50 ± 40.03; G4 182.00 ± 40.40; and G5 235.36 ± 14.28. We identified a very significant difference between G1 and G3 (*p* < 0.0001) and a significant difference (*p* < 0.05) between G1 and G2; G1 and G4; and G3 and G5, with G5 remaining longer in the closed arm.

The average time at the center for G1 was 31.86 ± 20.18; G2 74.85 ± 28.37; G3 66.69 ± 19.53; G4 60.55 ± 10.46; and G5 60.55 ± 23.65. There was no significant difference between experimental exercise groups and the sedentary control group concerning the center. However, there was a significant difference between groups G1 and G2 (*p* < 0.05).

### Discussion

Regarding the groups’ results in the open arms, G1 (aerobic, pre- and post-induction) and G3 (aerobic, post-induction), although some groups were submitted to the same type of exercise, presented different results. G1 had a significantly shorter mean time spent in the open arms than G3. This fact evidenced a behavior contrary to what was expected, which was a reduction in anxiety/stress-like behavior in both groups, not just in one.

A possible determinant of this result in our study would be triggering a defensive response and risk assessment behavior in the animals chronically exposed to exercise^14^, once G1 had a 14-week physical activity schedule compared to the seven weeks of G3. Given that both groups were under the same conditions in which the test was performed, differing only about the physical activity schedule, it can be highlighted that swimming practice can determine different responses on the behavior of rodents, according to the period of exposure^15^.

Another factor to consider is that the swimming activity for the mice is a forced test in which the animal seeks escape instead of spontaneous swimming, triggering an increase in plasma corticosterone catecholamines and glucose response. However, the literature points to the forced swimming test on a short schedule to reduce anxiety in these animals, in protocols ranging from one repetition to 15-day protocols of swimming exercises^16^^-^ 18.

Exposure to stressful stimuli triggers an adaptive physiological response that results in anxiety and stress behavior. This response involves different areas of the nervous system: the amygdala, the hypothalamic-pituitary-adrenal axis, and the locus coeruleus. If an organism cannot adapt to the stress response, it becomes, instead of an adaptive physiological response, a harmful pathological response^19^^,^ 20.

Physical exercise, especially aerobic activities, has antidepressant and anxiolytic effects and protects against deleterious consequences of stress. The positive effects of physical exercise are due to the reduction in the activation of central areas discussed before through neuroendocrine signals such as BDNF^19^^,^ 21.

In our data, G3 showed a significant difference (p < 0.5) when compared to G5 (sedentary). This result corroborates the effect of physical exercise on the control of anxiety in rodents already described. On the other hand, the group of sedentary animals remained exposed only to stressors, such as those derived from colitis and neoplastic evolution in the colon.

DSS-induced colitis in rodents increases plasma levels of corticosteroids and pro-inflammatory cytokines, such as elevation of interleukin-6 and GRO-alpha in the brain. Consequently, this response determines signals in the central nervous system, triggering a reflex stress response and anxiety-like behavior, which worsens with weight loss and progressive debilitation derived from the neoplasm^22^. Therefore, swimming activity positively affected anxiety-like behavior in our study. However, the time of exposure to the activity may have been a limiting factor since, given the same conditions between G1 and G3, only differing concerning the time of exposure to the activity; only G3 showed a significant difference concerning the control (sedentary group).

G2 (pre- and post-induction) and G4 (post-induction), representatives of the stair-climbing modality, did not show significant differences with the other groups regarding changes in anxiety-like behavior. In the graphical analysis, we can see a similarity between both groups, with only slight differences between the length of stay in each compartment of the elevated plus maze (ECL). However, when analyzing G2 and G3, comparing them with G5 (sedentary group), there was a difference concerning the time spent in the open arms, although there was no statistically significant difference. It is necessary to emphasize that the exercise of climbing a vertical ladder requires manipulation of the animal, such as placing it on the ladder, in addition to a gradual load, which may be a factor that interfered in our results, given that the literature reports that this manipulation of the rodent can be an interference factor on the ECL test^14^^,^ 23 ^,^ 24.

Although the CRC tumor induction model used in our study is similar to the one used in studies found in the literature, no analysis was found that used DSS as part of tumor induction and assesses the anxiety-like pattern in animals with cancer colorectal submitted to physical exercise.

The hypothesis of this study was directed to the effects of physical exercise in animals with CRC induced by AOM and DSS. Despite this, future studies with animals, not CRC induced, are necessary. Thus, the isolated effect of physical exercise on healthy animals can be individualized and analyzed between groups with CRC/sedentary, CRC/physical activity, healthy/sedentary, and healthy/physical activity.

## Conclusions

Aerobic exercise for seven weeks after tumor induction showed less impact on the animals’ behavior. On the other hand, it significantly increased the animals’ stress level when applied for 14 weeks before and after tumor induction.

The pre- and post-induction or only post-induction resistance exercise groups showed no difference in behavior between them. Still, there was a slight improvement in reducing stress/anxiety-like behavior concerning the control (sedentary group).
