# ERRATA

**DOI:** 10.1590/acb370508.errata

**Published:** 2023-03-13

In the manuscript **Assessment of stress and anxiety in mice with colorectal cancer submitted to physical exercise**, which DOI is: https://doi.org/10.1590/acb370508, published in the journal **Acta Cirúrgica Brasileira**, 2022;37(05)e370508, page 5, in the axis X of Figure 5:

**Instead of**:

Open Arm

Open Arm

Open Arm

**Should be**:

Open Arm

Closed Arm

Center

